# The Putative Involvement of Bacterial Symbionts in Cantharidin Biogenesis: An Explorative Study in Meloidae Insects

**DOI:** 10.1007/s00248-025-02683-1

**Published:** 2025-12-23

**Authors:** Arianna Basile, Lucrezia Spagoni, Daniela Visaggio, Filippo Pasquale Riggio, Marco Alberto Bologna, Emiliano Mancini, Paolo Visca, Alessandra Riccieri

**Affiliations:** 1 Department of Science, Roma Tre University, Rome, Italy; 2 Department of Integrative Marine Ecology, Stazione Zoologica Anton Dohrn, Calabria Marine Centre (CRIMAC), C.da Torre Spaccata, Amendolara, Italy; 3 NBFC, National Biodiversity Future Center, Palermo, Italy; 4 Department of Biology and Biotechnology C. Darwin, Sapienza University of Rome, Rome, Italy; 5 Santa Lucia Foundation IRCCS, Rome, Italy

**Keywords:** Bacteria, Cantharidin, Holobiont, Insect, Meloidae, 16S metataxonomic profiling, Terpene

## Abstract

Insect-microbes holobionts integrate host and microbial functions, with symbionts supporting nutrition, immunity, and defence, while producing metabolites, including beetle-derived compounds with therapeutic potential. Cantharidin is a toxic terpene produced by blister beetles (Coleoptera: Meloidae), endowed with defensive and pharmacological properties. Male insects produce and contain cantharidin in large quantities and transfer it to females upon mating. This study is aimed to gain information about the involvement of insect-associated bacteria in cantharidin biogenesis. To support the possibility that bacteria participate in cantharidin biogenesis, cantharidin antibacterial activity was assessed against six reference strains of representative species of *Bacillota* and *Pseudomonadota* from publicly available culture collections. All bacterial strains tolerated concentrations up to 600 µg/ml cantharidin in a standard antibacterial susceptibility test. To identify candidate bacterial lineages, 16S rRNA metataxonomic profiling of the V5–V6 region was performed in males and females from different Meloidae subfamilies and tribes. Analysis of the insect-associated microbiomes of the five cantharidin-producing species (*Lydus trimaculatus*, *Meloe proscarabaeus*, *Mylabris variabilis*, *Hycleus polymorphus*, *Zonitis flava*) revealed communities dominated by *Pseudomonadota*, with secondary contributions from *Actinomycetota* in *Z. flava* and *M. proscarabaeus* and *Cyanobacteriota* in the other host insects. Although overall community structure and composition did not differ significantly between sexes, a few taxa displayed consistent male-associated patterns, with *Staphylococcus*, *Cutibacterium* and one *Enterobacteriaceae* ASV resulting more abundant in males across all species. The intrinsic bacterial resistance to cantharidin, with both quantitative and qualitative differences in microbiome structure between male and female insects, makes the hypothesis of a putative involvement of bacteria in cantharidin biogenesis still viable.

**Supplementary Information:**

The online version contains supplementary material available at 10.1007/s00248-025-02683-1.

## Introduction

Insects and their microbial partners form holobionts, functioning as unified ecological and evolutionary entities [1]. Within this framework, symbiotic microorganisms play crucial roles in processes such as nutrient acquisition, immune regulation, and chemical defence, ultimately influencing insect physiology, ecology, and evolution [2, 3]. While microbes gain diverse benefits from their insect hosts, they in turn provide essential metabolites, such as nutrients and enzymes, supporting host growth, development, and reproduction [4]. Interestingly, some beetle-associated bacteria are involved in the biogenesis of defensive secondary metabolites [5], some of which are endowed with therapeutic properties [6]. For instance, *Dendroctonus frontalis* (Zimmermann, 1868) (Curculionidae: Scolytinae) engages a symbiosis with *Streptomyces* (*Actinomycetota*) producing the antifungal compound mycangimycin [7] that prevents the detrimental effects of the fungus *Ophiostoma minus* on its larval development [8–10]. Mycangimycin also shows a similar efficacy as other clinical antimalarial drugs, such as artemisinin and chloroquine, against *Plasmodium falciparum* [11]. Likewise, more than 35 *Paederus* species (Staphylinidae) contain pederin, a defensive toxic amide and a potent antitumor agent [12], which is produced through an intimate endosymbiotic relationship between these rove beetles and *Pseudomonas* spp. [13, 14]. A high prevalence of pederin-producing bacteria was found in females of *Paederus fuscipes* that, in fact, accumulate larger amounts than males of this terpene in their hemolymph [12]. In these examples, the collective genetic repertoire of the hosts and their associated microbes, known as the hologenome, represents the functional basis of the partnership and contributes to the adaptive potential of the holobiont.

Meloidae, or blister beetles, comprise about 3,000 species [15, 16] and are best known for producing cantharidin, a toxic terpene that circulates throughout their hemolymph, and is then exuded in yellowish oily droplets from legs and antennal joints as a defensive strategy [17]. Cantharidin has therapeutic properties [18, 19], and has been widely employed in the past as an aphrodisiac and antiphlogistic. Currently, it is used in the topical treatment of warts [20] and, owing to cytotoxicity, it also holds promise as an anticancer agent [21].

Cantharidin is endogenously generated in blister beetles, but its biosynthetic pathway has not yet been fully elucidated. It is known to be a by-product of the juvenile hormone, which is synthesized via the mevalonate pathway [22], a well-characterized metabolic route involved in the production of sesquiterpenes [23]. Within this pathway, farnesol is a key intermediate. Following its synthesis, farnesol undergoes a series of enzymatic oxidations leading to farnesoic acid, which is then converted into juvenile hormone acid and subsequently into juvenile hormone acid diol [24]. It has been suggested that some cytochromes P450 (CYP450s) may function in a not-yet-clarified manner in the cantharidin final biosynthetic step, converting the juvenile hormone acid diol into cantharidin [23, 25, 26]. Within bacteria, CYP450s are known to play important roles in many biosynthetic and biodegradative processes, providing a wide range of secondary metabolites and chemical transformations [27]. Although endogenous CYP450s may provide the final step of cantharidin biosynthesis in Meloidae, the prominent involvement and diversity of the bacterial CYP450 enzymes in terpene biosynthesis suggests that a bacterial contribution cannot be overlooked, also in line with what observed in other terpene-producing beetles [12]. These considerations highlight the importance of exploring if the hologenome may represent a functional unit in the processes underlying cantharidin biosynthesis.

Although cantharidin is produced by both male and female Meloidae individuals, it is worth noting that males exhibit consistently higher cantharidin levels [24]. Indeed, males produce cantharidin in large quantities and store it in the accessory glands of their reproductive organs, while females mainly receive it from males as a nuptial gift upon mating. After oviposition, females spread cantharidin on eggs likely to protect them from potential predators and/or parasites [19].

Given the sex-biased cantharidin production, the microbiome-mediated contribution to cantharidin biosynthesis is potentially identifiable through comparative analysis of the microbiota between sexes. If present, cantharidin-producing bacteria are expected to be conserved across Meloidae, and to be more abundant in males. Despite the sex-based differences in cantharidin biosynthesis in blister beetles [17], the possible involvement of bacterial symbionts in cantharidin biogenesis has not been investigated thus far. Moreover, the bacterial susceptibility to cantharidin has not yet been fully clarified [28, 29].

In this work, we first aimed at assessing bacterial susceptibility to cantharidin. A strong antibacterial activity of cantharidin would argue against its bacterial origin, since such a property could be harmful to potential producers. Conversely, the lack of antibacterial effects could be consistent with the involvement of the insect-associated microbiome in cantharidin biosynthesis. Then, we sought to describe the bacterial community dwelling or colonizing blister beetles. The antibacterial activity was tested against six prototypic bacterial species, while the insect-associated microbiome was investigated on a total of sixty specimens belonging to five species of blister beetles and both sexes. The differences in microbiome composition between males and females were inspected to identify sex-associated bacterial lineages as candidates for cantharidin-producing symbionts.

## Methods

### Assay for Bacterial Susceptibility to Cantharidin

Cantharidin (Sigma Aldrich) was freshly prepared as a 30 mg/ml stock solution in dimethyl sulfoxide (DMSO, Sigma Aldrich).

The cantharidin antibacterial activity was evaluated against six reference strains obtained from type culture collections of representative bacterial species, namely *Bacillus subtilis* subsp. *spizizenii* ATCC 6633, *Enterococcus faecalis* ATCC 29,212, and *Staphylococcus aureus* ATCC 25,923 among *Bacillota*; *Acinetobacter baylyi* ATCC 33,304; *Escherichia coli* MG1655 and *P. aeruginosa* ATCC 15,692 among *Pseudomonadota*.

The endogenous cantharidin concentrations are variable and poorly defined among Meloidae family members. Indeed, quantitative assessments of cantharidin in hemolymph of two species, *Lydus trimaculatus* and *Mylabris variabilis*, revealed cantharidin concentrations of 790 µg/g and 260 µg/g, respectively [17]. Considering that the insect hemolymph has a density ranging from approximately 1.03 to 1.18 g/ml [30], the cantharidin concentrations in antimicrobial susceptibility tests were set between 150 and 600 µg/ml to approximate the physiological conditions encountered by microorganisms within the hemolymph of Meloidae beetles.

For cantharidin antibacterial susceptibility testing, bacteria were grown overnight at 37 °C in Luria Bertani broth (LB, Acumedia), washed with saline solution, and diluted to OD_600_ = 0.001 (corresponding to ~ 5 × 10^5^ CFU/ml) in 96-well microtiter plates containing a final volume of 100 µl of cation-adjusted Mueller Hinton Broth (CAMHB, Becton Dickinson) per well, supplemented with increasing cantharidin concentrations (two-fold dilutions). The microtiter plates were incubated at 37 °C for 24 h. A well containing 2% DMSO in CAMHB, equivalent to the DMSO concentration in the wells containing the highest amount of cantharidin, was used as growth control.

Microbial growth was measured every two hours at OD_600_ using a Spark 10 M (Tecan) microplate reader.

## Selection of Specimens for Microbiome Analysis

**Fig. 1:**
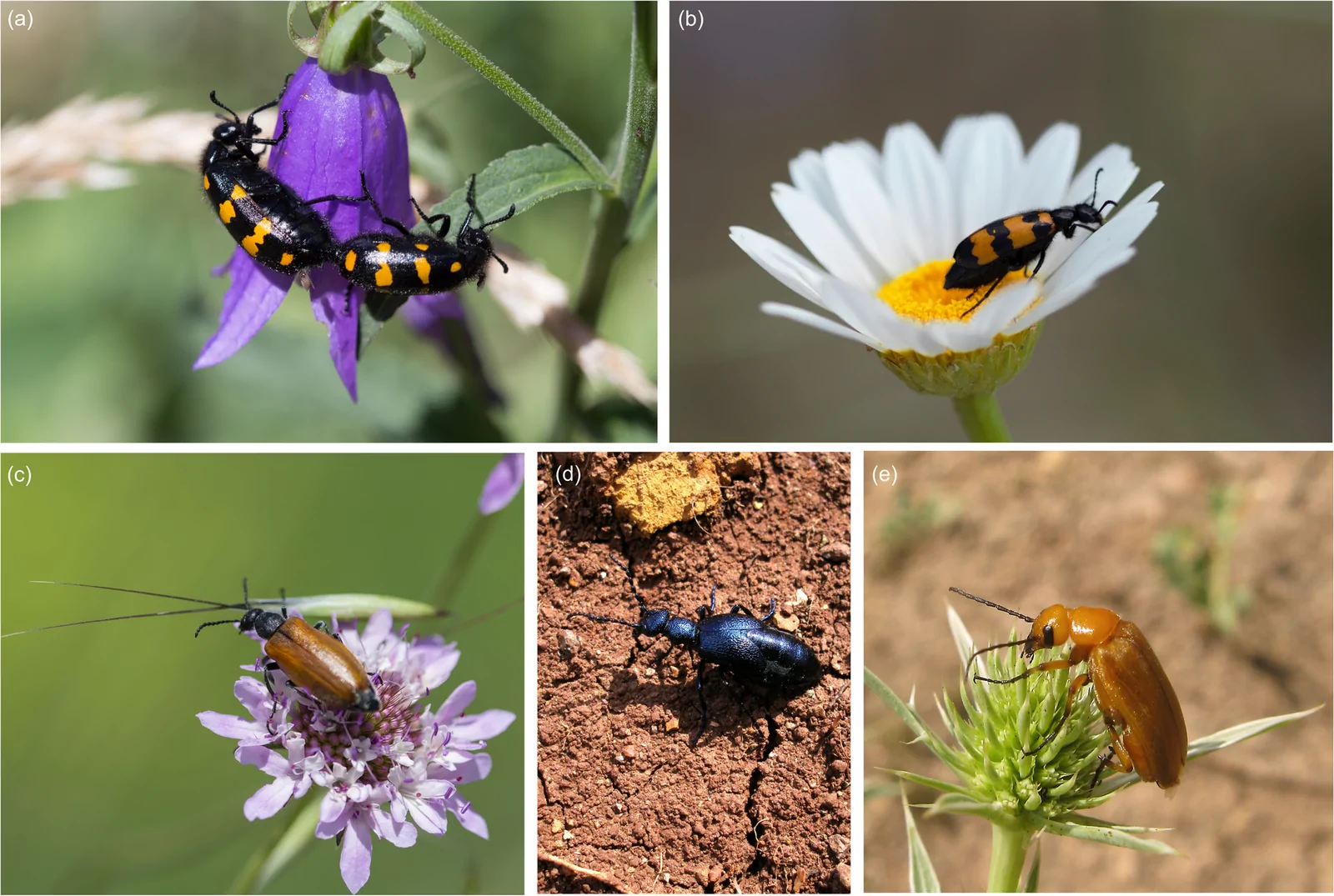
Habitus of the species considered for the whole microbiome analysis. *Hycleus polymorphus* (**a**), *Mylabris variabilis* (**b**), *Lydus trimaculatus* (**c**), *Meloe proscarabaeus* (**d**) and *Zonitis flava* (**e**)

The whole microbiome composition was determined for five representative species of Meloidae of different subfamilies and tribes, namely *Lydus trimaculatus* Kaszab, 1952 (Meloinae: Lyttini); *Meloe proscarabaeus* Linnaeus, 1758 (Meloinae: Meloini); *Mylabris variabilis* (Pallas, 1782), *Hycleus polymorphus* (Pallas, 1771) (Meloinae: Mylabrini); *Zonitis flava* Fabricius, 1775 (Nemognathinae: Nemognathini) (Fig. 1).

Two populations for each species were sampled, and six male and six female individuals for each population were tested. Overall, 60 specimens were included in the analyses, collected between 2015 and 2021 in different localities in Italy (Table S1). Insects were preserved in 96% ethanol at 4 °C and stored at Roma Tre University. Species and sex were determined using an Olympus SZX12 Stereomicroscope.

### DNA Extraction and Illumina Sequencing

DNA was extracted from the whole body (with no surface sterilization; [31]) using the DNeasy Blood and Tissue kit (Qiagen). The DNA concentration was measured using Nanodrop (Thermo scientific), to ensure that the DNA quantity was above 600 ng/µL. The V5-V6 region of the bacterial 16 S rRNA was targeted using the primer pair 783 F and 1027R and sequenced using the Illumina MiSeq v3 technology 300 bp by the Department of Earth and Environmental Sciences of Bicocca University in Milan.

### 16 S rRNA Gene Amplicon Sequence Analysis

Raw paired-end 16 S rRNA amplicon sequencing reads were processed using the DADA2 pipeline (v 1.34.0) [32] in R. Initial quality assessment was performed with *plotQualityProfile*, then sequences were filtered and trimmed using *filterAndTrim* with truncation lengths of 250 bp (forward) and 200 bp (reverse), a maximum expected error of 2, no ambiguous bases allowed, and PhiX removal enabled. Error correction was performed separately on forward and reverse reads using the error-learning method of DADA2 (*learnErrors*). Erroneous sequences were identified and removed using the *dada* command in pooling mode. Paired-end reads were merged based on sequence overlap with the *mergePairs* function to obtain complete amplicon sequences, and chimeric sequences were identified and removed. Taxonomic assignment was performed using the SILVA database (v. 138.1) [33].

A phyloseq object was constructed by integrating ASV counts, sample metadata, and taxonomy. The dataset was rarefied to retain 428 reads per sample. Bray-Curtis dissimilarities and weighted UniFrac distances were calculated from ASV abundances, followed by Principal Coordinates Analysis (PCoA) for beta diversity visualization, and differences in microbial community composition between groups were assessed using PERMANOVA as implemented in the function adonis2 of the R package vegan (v. 2.7-1).

Shannon alpha-diversity indices were calculated on the rarefied dataset using the *estimate_richness* function and plotted with ggplot2 (v. 3.5.1). Differences in microbial community richness between groups were assessed using the Wilcoxon Mann Whitney test. The software Functional Annotation of Prokaryotic Taxa (FAPROTAX) (v. 1.2.9) [34] was used to infer the potential metabolic functions of the identified ASVs.

The differential abundance of ASVs between sexes was assessed in the five insect species with DESeq2, using the Wald test and correcting p-values with the false discovery rate procedure [35]. To identify ASVs more abundant in male than female individuals, the difference in relative abundance was calculated using the formula:

$$\:i=\frac{M-F}{M}$$

where *M* represents the mean relative abundance in males and *F* in females. Positive *i* values indicate enriched ASVs in male individuals. For both M and F, the minimum value was arbitrarily set to 3.89408 × 10^− 5^ (equivalent to 1/2,568 reads), to avoid *i* = undefined. The threshold represents the scenario in which a single read from one biological replicate maps to the ASV of interest. Accordingly, the value of 2,568 is obtained by multiplying the number of reads retained per sample (i.e., 428) by the maximum number of replicates per sex–species combination (i.e., six).

### Identification of Terpene Biosynthetic Genes in *Staphylococcus* and *Cutibacterium* Genera

High-quality genomes from the genera *Staphylococcus* and *Cutibacterium* were retrieved from the NCBI database, filtering only genomes designated as “Reference genomes.” The final dataset consisted of 64 and seven reference genomes of *Staphylococcus* and *Cutibacterium* spp., respectively. All genomes were functionally annotated using the eggNOG tool (v.2.1.7; database v.5.0.2) [36], and the resulting annotations were screened for genes involved in terpene metabolism. Genes belonging to four different key pathways annotated in KeGG database for the metabolism of terpenes were searched, namely the mevalonate pathway (M00095), the non-mevalonate pathway (M00849), the pathway of biosynthesis of trans-farnesol [24], and the pathway of biosynthesis of staphyloxanthin (M01038). In addition, all genomes were analyzed for the presence of biosynthetic gene clusters (BGCs) using the Antibiotics & Secondary Metabolite Analysis Shell (antiSMASH, v.8.0.0) [37] to identify secondary metabolite pathways potentially implicated in terpene biosynthesis.

## Results

### Bacteria are Tolerant to Cantharidin

Antibacterial susceptibility to cantharidin was tested in bacterial species from two major phyla: *Bacillota* (*Bacillus subtilis* subsp. *spizizenii* ATCC 6633, *Enterococcus faecalis* ATCC 29212, and *Staphylococcus aureus* ATCC 25923) and *Pseudomonadota* (*Acinetobacter baylyi* ATCC 33304, *Escherichia coli* MG1655, and *Pseudomonas aeruginosa* ATCC 15692), at concentrations of 150, 300, and 600 µg/ml.

**Fig. 2:**
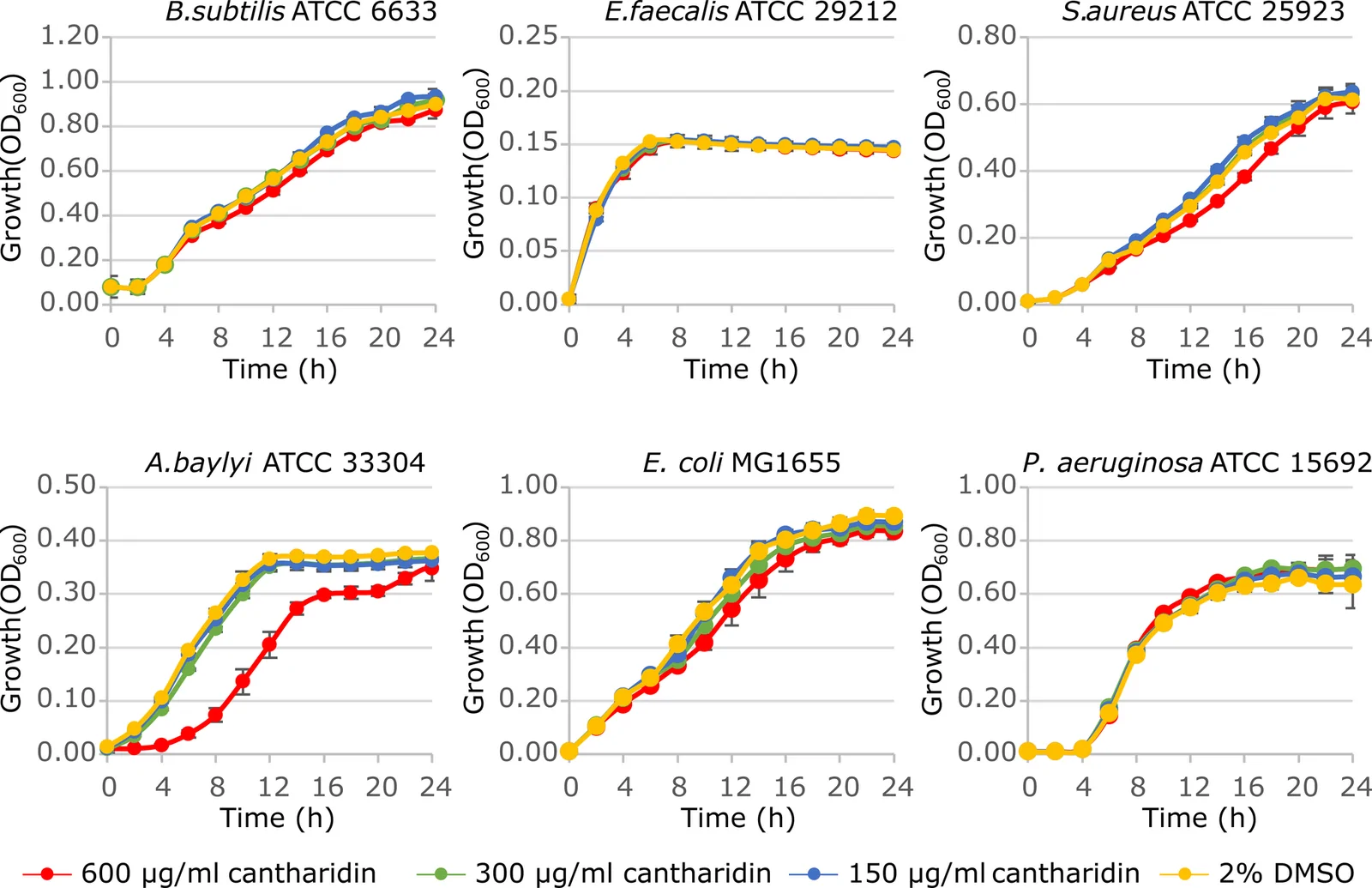
Effect of cantharidin on bacterial growth. Bacterial strains were inoculated at OD_600_ = 0.001 in CAMHB supplemented with 2% DMSO or the indicated cantharidin concentrations. Bacterial growth (OD_600_) was measured every 2 h for 24 h. Each value is the mean of two independent experiments ± the standard deviation. Media supplemented with 2% DMSO were used as growth controls without cantharidin

Growth profiling of the selected microorganisms showed that cantharidin causes no growth inhibition of all microbial species tested except the soil bacterium *A. baylyi* ATCC 33,304, which showed a slower growth rate in the presence of 600 µg/ml cantharidin, although reaching the same growth level as the untreated control after 24 h (Fig. 2).

Hence, the observation that cantharidin has no significant antibacterial activity would be in line with the hypothesis that its biosynthesis may be mediated or modulated by the insect-associated microbiome.

### Microbiome Profiling of Five Cantharidin-Producing Meloidae Species Revealed Few Male-Specific Signatures in the Bacterial Community

To investigate the hypothesis of a bacterial contribution to cantharidin biosynthesis, we characterised the whole body-associated microbiomes of 60 individuals, both males and females, of five blister beetle species. We aimed at identifying male-associated genetic signatures of bacterial taxa potentially involved in cantharidin biogenesis.

Amplicon sequencing targeting the V5-V6 hypervariable region of the 16 S rRNA was performed, and a total of 7,572,512 paired-end raw reads were obtained, averaging 126,208 reads per sample (Table S2). The raw data were processed using the DADA2 pipeline, as detailed in Materials and Methods, yielding 4,135 ASVs (916,512 reads). Two samples, namely *M_variabilis_*M_4943c and *L_trimaculatus*_F_4195a, were excluded due to a low number of merged reads (55 and 61 reads, respectively). ASVs were then rarefied, retaining 428 reads per sample, resulting in 2,002 ASVs on a total of 24,824 reads (Table S3).

**Fig. 3:**
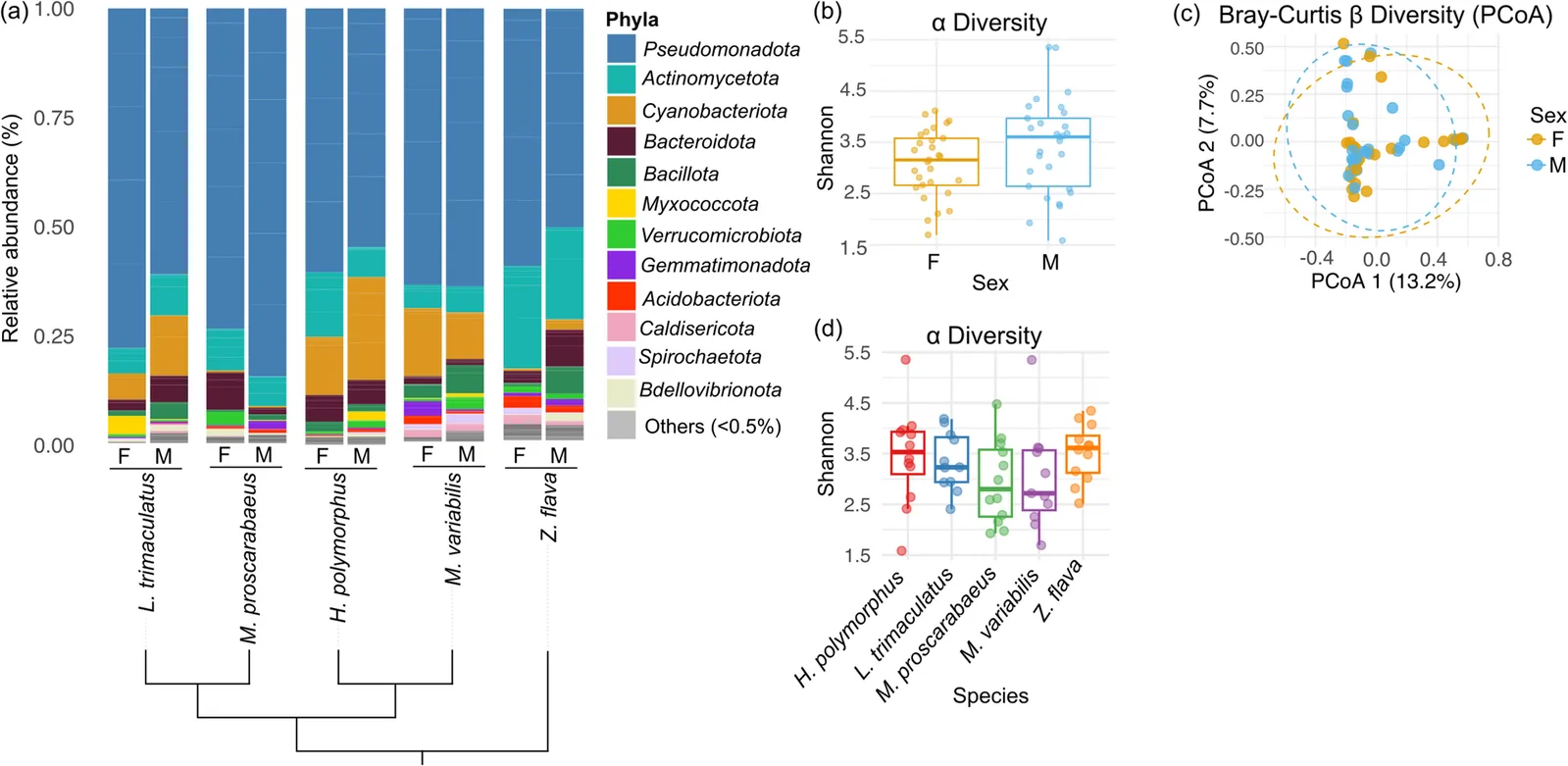
Analysis of the bacterial communities in five insect species of the Meloidae family. (**a**), Microbial community composition inferred from 16S rRNA gene amplicon sequencing (V5-V6 region). Phyla showing 0.5% relative abundances were grouped and are displayed as “Others (< 0.5%)”. The relative abundance values are provided for each blister beetle species. The data represent mean values derived from five biological replicates for *M. variabilis* males and *L. trimaculatus* females, and from six biological replicates for all eight- remaining sex-species combinations. The cladogram reflects the phylogenetic relationships among Meloidae species [15]. (**b**), Box plots of the Shannon index of diversity with samples grouped by sex. Statistical differences were calculated using the Wilcoxon test, and the p-values were adjusted with the FDR method. (**c**), beta-diversity plot based on Bray-Curtis distances, with sorting determined by PCoA. Dotted ellipses delimit samples of the same sex (**d**): Box plots of Shannon index of diversity with samples grouped by insect species. Statistical differences were calculated using the Wilcoxon test, and the p-values were adjusted with the FDR method

In all five Meloidae species, the microbial community is dominated by bacteria belonging to the phylum *Pseudomonadota*. The second most abundant phyla are *Actinomycetota* in *Z. flava* and *M. proscarabaeus*, and *Cyanobacteriota* in *H. polymorphus*, *L. trimaculatus*, and *M. variabilis* (Fig. 3a).

At a finer taxonomic resolution, insect-associated microbial communities showed species-specific differences. Members of the *Enterobacteriaceae* family were predominant in *L. trimaculatus* and *M. variabilis*, suggesting a shared microbial niche or functional role (Fig. S1). A bacterial community primarily composed of *Rickettsiaceae* was detected in *H. polymorphus*, while *M. proscarabaeus* displayed a more heterogeneous composition of the bacterial taxa, including *Erwiniaceae*, *Hafniaceae*, and *Comamonadaceae* (Fig. S1). Bacterial community composition shows no apparent relationship with the established phylogeny of Meloidae (Fig. 3a). The distinct microbiome structures denote divergent ecological associations and potentially unique microbial functions in the host species. Nonetheless, functional category analysis across samples (Fig. S2) suggests that fermentation and chemo-organotrophy are the central energy pathways in the bacterial communities colonizing the five blister beetle species (Table S3).

Since males of Meloidae produce higher cantharidin levels than females, inter-sexual differences were more in-depth investigated. Despite the marked interspecific differences in bacterial colonization profiles of the insects, both alpha and beta diversity (measured both as Bray-Curtis and Weighted UniFrac) analyses showed no significant differences between male and female individuals of the same species, suggesting that microbial community composition and structure are similar between sexes (Fig. 3b, c; Fig. S3). Similarly, alpha diversity did not differ significantly between Meloidae species (Fig. 3d).

Overall, genus *Cutibacterium* was more abundant in males than in females in all insect species except *Z. flava*, while genus *Staphylococcus* was absent in females and present only in males of all species. These differences may suggest, though they do not establish, a possible link between these genera and cantharidin synthesis (Table S4).

**Fig. 4:**
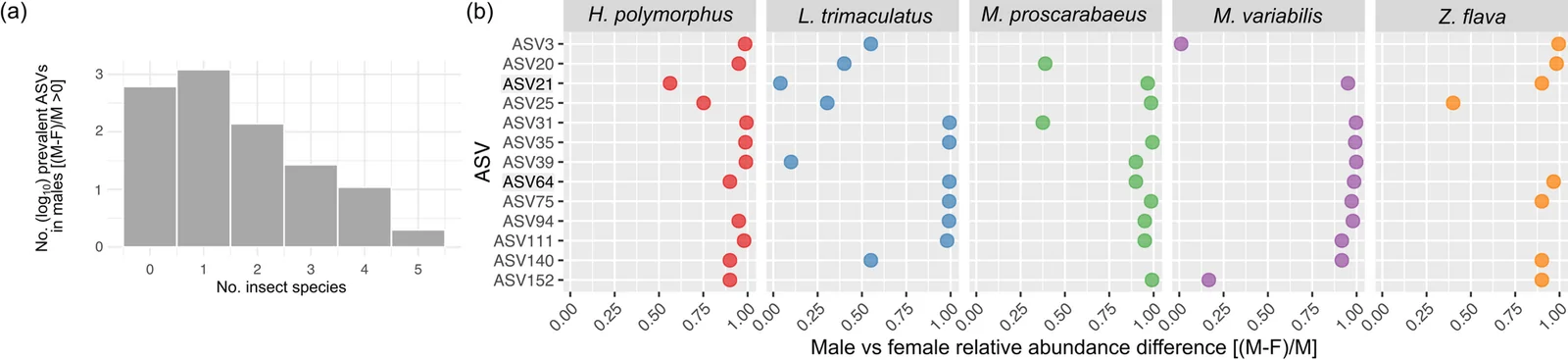
Overview of the prevalent ASVs in male individuals of Meloidae beetles (**a**), Bar plot showing the number of ASVs that are more prevalent in males for each species (*H. polymorphus*, *L. trimaculatus*, *M. proscarabaeus*, *M. variabilis*, *Z. flava*), calculated using the formula (M-F)/M, where M and F represent the relative abundances in male and female individuals, respectively. The ASV counts are shown on a log₁₀ scale for each meloid species. (**b**), List of ASVs which are more abundant in male than female individuals in at least four out of five meloid species (*H. polymorphus*, *L. trimaculatus*, *M. proscarabaeus*, *M.variabilis*, *Z. flava*). For each ASV the differential abundance between male and female individuals is reported only when > 0. The more abundant ASVs in male than female individuals in all five species (ASV21 and ASV64) are in bold

Then, we focused on ASVs that were more abundant in male than female individuals of all five Meloidae species. The differential abundance analysis of bacterial ASVs between male and female individuals of the same species, performed using the Wald test, did not reach statistical significance in any insect species. Therefore, the difference in abundance of each ASV between sexes was expressed as *i* index (see Materials and Methods), a descriptive measure of differential abundance. For each ASV, the *i* index was calculated five times (one for each meloid species). For *i* values > 0, the ASV is more abundant in male than female individuals for a given insect species. Therefore, each ASV can be more abundant in males than females (*i > 0*) in none, one, two, three, four, or all the sampled Meloidae species (Fig. 4a).

Apart from 616 ASVs endowed with *i* ≤ 0 in all meloid species, 1,207 ASVs were more abundant in males than in females in only one species, 139 in two, and 27 in three of the five meloid species (Fig. 4a; Table S5). Eleven taxonomically heterogeneous ASVs, encompassing different bacterial genera and families, were more abundant in males than females in four out of five meloid species (Fig. 4a, b; Table S5). Only two ASVs were consistently more abundant in male than female individuals across all five meloid species, namely ASV64 and ASV21 (Fig. 4a, b; Table S5), referable to the members of *Cutibacterium* genus and the *Enterobacteriaceae* family, respectively.

Thus, while sex-dependent differences in microbiome composition between Meloidae species were observed, only a few bacterial signatures were more prevalent in male than female individuals in most or all species.

### Terpene Biosynthethic Gene Clusters in *Staphyloccus *spp. and *Cutibacterium *spp.

The comparative analysis of available reference genomes of the representative *Cutibacterium* spp. and *Staphylococcus* spp. revealed that biosynthetic gene clusters (BGCs) involved in terpene and terpene-precursor biosynthesis were widespread in these bacterial genera, with every genome analysed containing at least one cluster (Fig. S4a, b). In some cases, AntiSMASH predicted a specific terpene product, for example staphyloxanthin, but the end-product of many other BGCs was unassigned.

The comparative analysis of gene annotations unveiled that *Cutibacterium* spp. possess the non-mevalonate (methylerythritol phosphate, MEP) pathway for the biosynthesis of isopentenyl pyrophosphate (Fig. S4c), whereas *Staphylococcus* spp. carries the genes for both the mevalonate-dependent (MVA) and the MEP pathway for terpene biosynthesis. In addition, both genera harbour the gene annotated as K13787, implicated in the biosynthesis of farnesol, an essential intermediate in juvenile hormone production.

## Discussions

Cantharidin is a bioactive terpenoid produced by blister beetles and implicated in both defence and reproductive behavior. The compound has a long-standing history of use in both folk and traditional medicine. It was employed in traditional Chinese medicine as well as by ancient Greek and Roman physicians for a variety of purposes, ranging from the treatment of warts to potential anti-cancer therapies, as an antiphlogistic, and even as an aphrodisiac by different human populations [19, 38]. Despite its historical background, the actual clinical use of cantharidin is limited due to its toxicity, particularly affecting the renal, hepatic, urinary, and gastrointestinal systems [20]. Approved medical applications are restricted to topical treatments for warts and for molluscum contagiosum, a viral skin infection [39]. Numerous studies have highlighted its promising broad-spectrum antitumor activity against various types of cancers, including leukemia, esophageal carcinoma, lung cancer, rectal cancer, and breast cancer, with notable results reported for liver cancer [40].

The mechanisms of cantharidin activity in tumour cells involve the modulation of multiple signaling pathways regulating cell proliferation, apoptosis, autophagy, cell migration, and invasion [40]. These effects are primarily mediated through its potent inhibition of serine/threonine protein phosphatases PP1, PP2A, and PP5, which are key regulators of eukaryotic cell fate [41–43]. While the antitumor properties of cantharidin have been quite widely studied, its effect(s) on bacteria have never been fully clarified. In this study, we demonstrated that cantharidin does not exert growth inhibitory activity against representative bacterial strains from distant phyla endowed with extremely diverse cell structures, including both Gram-positive and -negative species, namely *Bacillota* (*B. subtilis* subsp. *spizizenii* ATCC 6633, *E. faecalis* ATCC 29212, and *S. aureus* ATCC 25923) and *Pseudomonadota* (*A. baylyi* ATCC 33304, *E. coli* MG1655, and *P. aeruginosa* ATCC 15692). This lack of activity could be explained by fundamental differences in the structure of prokaryotic phosphatases. Although bacterial phosphatases catalyze similar reactions, they typically belong to distinct protein families (e.g., PPM-type phosphatases) and exhibit different structural and regulatory features compared to their eukaryotic counterparts [44]. These structural differences at the target level are likely to account for the apparently intrinsic resistance of the tested bacteria to cantharidin. Moreover, exclusion by the bacterial membrane or extrusion by the activity of efflux pumps could also contribute to cantharidin resistance. Since cantharidin susceptibility was determined on a definite panel of reference strains under standard conditions for antimicrobial susceptibility testing, more focused studies on bacterial isolates from Meloidae insect are needed to confirm that cantharidin tolerance is an intrinsic feature of the bacterial component of the Meloidae microbiota. Moreover, the standard antimicrobial susceptibility test may not reflect the complex insect-microbe interactions and overlooks the potential metabolic activities of the holobiont that could affect cantharidin susceptibility in vivo.

The lack of cantharidin antibacterial activity is a prerequisite for the plausibility of the hypothesis that the insect-associated microbiome may play a role in its biosynthesis. This hypothesis is consistent with the evidence that insect-associated bacterial symbionts can contribute to the synthesis of essential nutrients or bioactive compounds involved in defense against predators [45]. Since males of the Meloidae family produce more cantharidin than females, we investigated the microbiome of representative species of the family, belonging to the main phylogenetic lineages, namely *H. polymorphus*,* L. trimaculatus*,* M. proscarabaeus*,* M. variabilis*, and *Z. flava*. Our results revealed that the most abundant phylum in all the samples was *Pseudomonadota*, in line with previous studies on coleopteran species such as scarab beetles (*Anomala dimidiata* (Hope,1831)), ground beetles (*Harpalus rufipes* (De Geer, 1774)), and wood-boring beetles (*Monochamus saltuarius* (Gebler, 1830)), where *Pseudomonadota* was identified as a dominant component of the gut microbiota [46–48]. The prevalence of this phylum may reflect its metabolic versatility and potential role in nutrient assimilation, degradation of plant-derived compounds, and detoxification processes commonly required in phytophagous beetles [45]. Gut bacteria of the phylum *Pseudomonadota* have been shown to influence coleopteran biology in several systems. These bacteria can breakdown recalcitrant plant polymers by producing cellulases (e.g., *Pseudomonas* sp. [49]). or pectinases (e.g., *Stammera* sp. [50]). They also contribute to the degradation of plant toxins, as observed for *Pseudomonas fulva* (caffeine; [51]), *Acinetobacter* sp. (tea saponin [52]), and *Novosphingobium* sp. (the phenolic compound naringenin [53]).

Although microbial communities in Meloidae exhibit clear species-specific structures, with dominant taxa varying across species, overall diversity did not differ significantly between sexes. However, *Cutibacterium* spp. and *Staphylococcus* spp. were predominant in males in the majority or even all of the insect species. Moreover, two ASVs, referred to *Cutibacterium* and *Enterobacteriaceae*, showed a trend toward higher abundance in males in all species, suggesting putative sex-associated microbial patterns. However, these patterns did not reach statistical significance, and their potential link to cantharidin biogenesis should be cautiously considered.

Members of the *Cutibacterium* and *Staphylococcus* genera have previously been detected in insect microbiomes [54–56], suggesting that they could play a role in host-associated microbial communities. Notably, members of these two genera are notoriously fatty acids-resistant colonizers of the human skin [57–59], and contain numerous biosynthetic gene clusters predicted to produce terpenes or terpene precursors, though most of their metabolic end-products are still uncharacterized. This raise the possibility that insect-associated *Cutibacterium* spp. and *Staphylococcus* spp. may contribute to the production of intermediates in the biosynthesis of juvenile hormone or cantharidin, potentially influencing host physiology and microbial interactions, but an experimental validation is required to uncover their functional roles and confirm an involvement in the biosynthesis of cantharidin.

Indeed, the biosynthetic pathway of cantharidin has not yet been fully elucidated. As for many other terpenoids, cantharidin biosynthesis is hypothesized to originate from the MVA pathway, a well-characterized metabolic route involved in the production of sesquiterpenes [24]. Within this pathway, farnesol is a key intermediate. Following its synthesis, farnesol undergoes a series of enzymatic oxidations leading to farnesoic acid, which is then converted into juvenile hormone acid and subsequently into juvenile hormone acid diol. These compounds are recognized as crucial intermediates in the biosynthesis not only of the juvenile hormone but also of cantharidin [24]. While early steps involving isoprenoid precursors have been characterized to some extent, the downstream transformations leading to cantharidin remain unresolved, suggesting the involvement of yet-unidentified insect enzymes or, possibly, microbial partners contributing to the final stages of its assembly. These considerations highlight the importance of the hologenome as a functional unit in elucidating the processes underlying cantharidin production.

In this study, microbiome analysis was performed using DNA from the whole insect body. However, this approach may overlook tissue- or organ-specific microbial contributions, given that cantharidin is not evenly distributed throughout the body but rather accumulates in specific tissues and organs. For instance, in *E. chinensis*, its localization displays a pronounced tissue-specific trend, with the male accessory glands containing over 70 times the concentration found in the head [60, 61]. The fat body showed the second-highest cantharidin content (approximately 2.11 mg/g), exhibiting the highest expression levels of the biosynthetic gene juvenile hormone epoxide hydrolase, suggesting it may be a key site of active biosynthesis. Therefore, targeting these tissues could enrich for microbial taxa that are more likely to participate in cantharidin production. Fat bodies have been demonstrated to host endosymbiont bacteria contributing to the host metabolism, as in the case of *Blattabacterium*, an endosymbiont of *Blattella germanica* (Blattodea) fat bodies, which participates in the synthesis of essential amino acids, nitrogen recycling, and modulates immune gene expression [62].

## Conclusions

In conclusion, our findings demonstrate that representative strains of major bacterial taxa are resistant to the toxic activity of cantharidin and provide novel insights into sex-associated differences in the Meloidae microbiome, but they do not yet offer sufficient evidence for a bacterial contribution to cantharidin biosynthesis. 16 S rRNA gene amplicon sequencing did not identify predominant bacterial taxa uniquely associated with male individuals, despite their consistently higher cantharidin levels. However, the observed differences in microbiome composition between males and females cannot exclude the hypothesis of a putative involvement of the insect hologenome in the biosynthesis of this terpenoid. More focused approaches are needed to evaluate if bacteria, including those identified in this study, have a role in cantharidin biogenesis. Future research should therefore be directed to the identification of the reactions and enzymes involved in the terminal steps of cantharidin biogenesis, and the functional characterization of the microbiome in producing organs (i.e., the fat bodies).

## Supplementary Information

Below is the link to the electronic supplementary material.

Supplementary Material 1 (XLSX 576 KB)

Supplementary Material 2 (DOCX 1.05 MB)

## Data Availability

Raw sequencing reads have been deposited to the NIH Sequence Read Archive under the bioproject number: PRJNA1327909.

## Declarations

### Author Statement

We sincerely thank the anonymous Reviewers for their insightful comments and constructive feedback. We have carefully considered their criticism and revised the manuscript to improve its clarity and readability. Additional statistical analyses have also been incorporated to reinforce the robustness of our findings.
