# Supplementary material for: The Putative Involvement of Bacterial Symbionts in Cantharidin Biogenesis: An Explorative Study in Meloidae Insects
Source: Microb Ecol. 2025 Dec 23;89:34. doi: 10.1007/s00248-025-02683-1 (PMC12816098; doi:10.1007/s00248-025-02683-1)
Supplement: Supplementary file 1 [file 248_2025_2683_MOESM2_ESM.docx]

**The putative involvement of bacterial symbionts in cantharidin biogenesis: an explorative study in Meloidae insects**

Arianna Basile^1*^, Lucrezia Spagoni^2*^, Daniela Visaggio^1^, Filippo Pasquale Riggio^1^, Marco A. Bologna^1,3^, Emiliano Mancini^3,4^, Paolo Visca^1,3,5^, Alessandra Riccieri^1,3^

^1^ Department of Science, Roma Tre University, Rome, Italy

^2^ Department of Integrative Marine Ecology, Stazione Zoologica Anton Dohrn, Calabria Marine Centre (CRIMAC), C.da Torre Spaccata, Amendolara, Italy

^3^ NBFC, National Biodiversity Future Center, Palermo, Italy

^4^ Department of Biology and Biotechnology C. Darwin, Sapienza University of Rome, Rome, Italy

^5^ Santa Lucia Foundation IRCCS, Rome, Italy

* Authors equally contributed

Correspondence: Arianna Basile [arianna.basile@uniroma3.it](mailto:arianna.basile@uniroma3.it); Alessandra Riccieri [alessandra.riccieri@uniroma3.it](mailto:alessandra.riccieri@uniroma3.it)

**Figure S1**. Family-level bacterial community structure in five insect species of the *Meloidae* family inferred from amplicon sequencing of the 16S rRNA gene (V5-V6 region). The ten most abundant bacterial families for each insect species were identified, and the bacterial families not belonging to any of these five lists are grouped as “Others”. The data for sex and species are average values of five biological replicates for *M. variabilis* and *L. trimaculatus*, and six biological replicates for *H. polymorphus*, *M. proscarabaeus*, and *Z. flava*.

**Figure S2**. Predicted metabolisms inferred from functional categories across the microbiome of male and female individuals of five insect species (*H. polymorphus*, *L. trimaculatus*, *M. proscarabaeus*, *M. variabilis*, *Z.* *flava*) species. Functional categories were inferred from FAPROTAX analysis. Each circle refers to a metabolic function. The color refers to the sex (F or M). The area of each circle is proportional to the relative abundance (%) of the functional category in the metagenomic sample.

**Figure S3**. Beta-diversity plot based on Weighted UniFrac distances, with sorting determined by PCoA; 95% confidence ellipses are shaded.

**Figure S4:** Biosynthetic gene clusters (BGCs) identified by AntiSMASH in *Cutibacterium* spp. (**a**) and *Staphylococcus* spp. (**b**). Overview of genes identified in *Cutibacterium* spp. (upper panel) and *Staphylococcus* spp. (lower panel) involved in terpene metabolism, namely the non-mevalonate pathway, mevalonate pathway, farnesol biosynthetic pathway, and staphyloxanthin biosynthetic pathway (**c**). Abbreviations: t3pks, type III polyketide synthase; ripp-like, other unspecified ribosomally synthesised and post-translationally modified peptide product; nrps, non-ribosomal peptide synthetase.
